# The Shortened Dental Arch Concept: Awareness, Knowledge, and Practice of Dentists in Dubai and the Northern Emirates, United Arab Emirates

**DOI:** 10.1155/2022/6018650

**Published:** 2022-11-02

**Authors:** Haleimah AlHmoudi, Amar H. Khamis, Haitham Elbishari, Fatemeh Amir-Rad

**Affiliations:** ^1^Mohammed Bin Rashid University of Medicine and Health Sciences, Hamdan Bin Mohammed College of Dental Medicine, Dubai Healthcare City, Dubai, UAE; ^2^Prosthetic Section, Khorfakkan Specialized Dental Center, Ministry of Health and Prevention, Dubai, UAE

## Abstract

**Methods:**

This is a cross-sectional study utilizing an online questionnaire anonymously to investigate the awareness and views of dentists about SDA. The questionnaire was sent to all 901 dentists registered with the Emirates Medical Association (EMA). The questionnaire consists of 17 questions, which comprise demographics, awareness, and application in dental practice, preferred treatment modality, and risks and benefits associated with SDA. The data were analyzed using SPSS Statistics.

**Results:**

The response rate reported was 40.3%. Two-thirds of the respondents (65.8%) were aware of the SDA concept; however, it was not usually applied in clinical practice (*n* = 196, 54.7%). Specialists were more aware of the concept (*p* ≤ 0.001) and applied it more frequently in their clinical practice (*p*=0.041) than general dental practitioners (GDPs). Respondents agreed that SDA was associated with the risks of teeth migration (*n* = 211, 59.9%), tooth wear (*n* = 196, 55.8%), and/or temporomandibular disorder (TMD) (*n* = 163, 45.3%). The implant was the treatment of choice for many of the participants (*n* = 169, 46.6%) to replace missing molars, followed by the acrylic removal partial denture (RPD) (*n* = 129, 35.5%).

**Conclusions:**

Most dentists who responded to this survey were aware of the SDA concept and had a positive attitude about it. However, they did not apply it frequently in their clinical practice.

## 1. Introduction

A normal healthy person with no developmental disorders develops a total number of 28 to 32 permanent teeth (the third molars may not always form or erupt) [1]. When several posterior teeth are missing, the dentist must take several variables into account when caring for partially dentate patients. Maintaining oral functionality, or masticatory ability, is one of the most critical elements to address, which leads dentists to wonder how many teeth are needed to suit a patient's functional needs [2]. Traditional restorative dentistry treatment planning is based on the morphological approach which suggests that in a broken-down dentition, as many teeth as technically possible should be saved or replaced. From this point of view, to meet oral functional needs, complete dental arches or at least 28 teeth were deemed necessary [3]. However, individuals' functional demands and the number of teeth required to meet them varies; hence, we should tailor our restorative care to each person's unique demands and adaptive capacity [2].

The problem-oriented method, developed in the 1980 s by the Dutch prosthodontist Arnd Kayser, is another way to establish a treatment plan for partially dentate individuals [3]. This functional approach focuses on maintaining a natural, functioning, and healthy dentition with sound biological criteria to provide the patient with satisfactory function and adaptive capacity [4, 5]. The shortened dental arch (SDA) is an example of this problem-oriented approach aimed to minimize complex restorative treatments. The SDA can be defined as “a dentition where the most posterior teeth are missing” [6].

In many cases, the replacement of all missing teeth is possible, keeping in mind the cost associated with and the real need for complete dental arches [7, 8]. Since the modern diet does not require a complete and functionally intact dentition, and occlusal stability and functional requirements can be met with the presence of the anterior and bicuspid teeth, it is debated that the replacement of lost molars is not necessary unless there is a functional and/or aesthetic requirement that justifies this replacement [1, 2, 9]. This means in certain cases, replacing missing molars with cantilevers, implant-supported prosthesis, resin-bonded bridge (RBB) or distal extension removable partial denture (RPD) can be considered as overtreatment [2].

The SDA concept focuses on providing partially dentate patients with the advantages of oral functionality, improved oral hygiene, and comfort, while avoiding overtreatment and its unnecessary costs and questionable benefits [2, 10]. The effect of SDA on patients' masticatory ability, signs, and symptoms of temporomandibular joint disorders (TMD), remaining teeth migration, oral comfort, and periodontal support has been investigated. Studies found no clinically significant differences between people with SDA and those with complete dental arches regarding the abovementioned criteria [4, 10–12]. These results indicate that the classical morphological approach to restoring all missing teeth and providing the patient with complete dental arches is not scientifically supported [11].

In 1992, the World Health Organization (WHO) stated that: “when it is not functionally or aesthetically necessary, and if occlusal disharmonies are not causing myofascial pain or problems of the temporomandibular joint, teeth should not be replaced” and “Prostheses that endanger the remaining dentition and/or supporting tissues are to be discouraged” [13]. Yet, many studies have shown that although the SDA is accepted by a great number of dentists, they do not always apply the concept in their practice [10, 14–19].

The application of the SDA concept among dentists in the United Arab Emirates (UAE) and patients' responses to this type of treatment has not yet been investigated. Therefore, the aims of this study were as follows:

1. Evaluate the awareness of dentists about SDA concept and its application in their practice.
2. Investigate the preferred treatment modality for SDA patients and the factors that affect this decision among dentists in the UAE.

## 2. Materials and Methods

### 2.1. Study Design and Participants

This was a cross-sectional study that collected information on dentists' awareness of SDA and whether they use it in their daily practice in the UAE. General dental practitioners (GDP) and dental specialists registered with the Emirates Medical Association (EMA) in UAE were eligible to participate. An online survey was shared by e-mail to a total of 901 dentists who registered under EMA. The EMA registered dentists were from multiple emirates and held Dubai Health Authority and/or Ministry of Health and Prevention license, allowing them to practice dentistry in Dubai and the Northern Emirates (Ras al-Khailmah, Sharjah, Ajman, Fujeirah, and Umm al-Quwain) of the UAE.

### 2.2. Ethical Approval

The study was approved by the institutional review board of Mohammed Bin Rashid University of Medicine and Health Sciences (MBRU-IRB-2020-020), and Ministry of Health and Prevention (MOHAP/DXB-REC/MMM/No. 47/2020).

### 2.3. Data Collection

A voluntary anonymous modified questionnaire used in a previous study in Saudi Arabia by Alammari [15] was sent through emails to all dentists registered in EMA. Permission was granted by Alammari to use her validated structured questionnaire [15]. The original questionnaire consisted of 6 demographic questions and 13 questions about SDA. The current questionnaire was altered to contain 6 demographic and 11 questions about SDA, excluding consent-related questions, which were not counted in the total number of questions. The differences between the original and the current questionnaire on demographic questions were limited to replacing the questions on nationality and location of practice with questions on the country of the last academic degree taken and years of experience. Moreover, in the SDA-related section, the two original questions regarding the number of cases treated based on the SDA concept and whether dentists will lose income if SDA is implemented have been removed. Hence, modifications were implemented to the current questionnaire, a small pilot study involving ten participants was conducted to ensure that participants understood the questions and to identify any issues with these questions.

A sample size was calculated based on adopting 95% power and 5% error. A representative sample size of 542 participants was calculated for inclusion. To account for possible nonresponse, a total of 901 EMA registered dentists were included.

An e-mail explaining the aim of the study and providing brief information about the SDA concept being a problem-oriented approach as described by Kayser was provided with the online questionnaire. Besides consent-related questions, the questionnaire consisted of demographic questions about gender, working sectors type, education level and specialty, years of experience, and country of last academic degree, followed by questions about dentists' knowledge of SDA, their use of the SDA concept and the treatment they typically provide to patients with pre-existing SDA conditions, the primary goal of treatment, dentists' attitude toward statements about SDA, and dentists' opinion regarding the benefits and drawbacks of SDA (supporting information, Appendix A). The data collection took place from 9 November 2020 till 28 February 2021. The first e-mail was followed by a reminder e-mail after 2 weeks and a second reminder after 2 months.

### 2.4. Statistical Analysis

Data were analyzed using Statistical Package for Social Sciences (SPSS) for Windows (IBM- SPSS) version 25.0 (SPSS Inc., Chicago, IL, USA). A measure of percentage was performed as descriptive statistics for categorical variables. The data were described and analyzed in contingency and frequency tables, means and standard deviations were calculated using the independent Student's *t*-test for analyses of groups of dentists with respect to gender, specialty, country of last academic degree, years of experience, and dental organization. To study explanatory patterns regarding the variables (gender, type of dental practice, specialty, country of last academic degree, and years of experience) influencing dentists' choice of treatment in an SDA and the frequency of SDA usage, a chi-square analysis was used. These categorical variables were cross-tabulated to examine the independency between variables. For such variables, the *χ*2-square test or Fisher's exact test, as appropriate, was used. A *p* value of less than 0.05 is considered significant in all statistical analyses.

## 3. Results

### 3.1. Attributes of the Responding Dentists

A total of 363 out of 901 recipients responded to the questionnaire, which accounts for a 40.3% response rate. There were more males (*n* = 239, 66.9%) among the respondents than females (*n* = 118, 33.1%). The majority of the respondents were UAE graduates (*n* = 204, 56.2%) and 36.1% (*n* = 131) were international graduates. It is important to note that the questionnaire permitted nonresponses, and as a result, the total number of responses for some questions did not equal 363.  Table 1 contains more information about the sample under investigation.

### 3.2. Awareness about SDA and Selected Mode of Treatment for SDA

Even though two-thirds of the dentists in the survey (*n* = 237, 65.8%) had heard of SDA, more than half of them did not use it (54.7%) or only used it sometimes (14.2%) in their practice. Those who preferred to replace missing molars chose implant-supported prosthesis (*n* = 169, 46.6%), followed by acrylic RPD (*n* = 129, 35.5%). The most common reason for replacing molars was to improve mastication (*n* = 160, 44.3%), followed by improving both mastication and aesthetics (*n* = 159, 44.3%) (Table 2). A substantial proportion (*n* = 123, 34.2%) of the surveyed dentists were unaware of the concept and came to know about it only when reading this survey.

### 3.3. Relationship between Different Factors and SDA Awareness and Mode of Treatment for SDA

#### 3.3.1. Highest Qualification

The findings demonstrated that specialists were more aware of the SDA idea and used it in clinical practice more frequently than GDPs (*p* ≤ 0.001, 0.041, respectively). Many GDPs (*n* = 87, 42.0%) were unaware of SDA when they received this poll. Also, many GDPs (*n* = 120, 57.4%) chose to replace the missing molars, whereas the majority of specialists (*n* = 88, 59.1%) recommended not to (*p*=0.001). When it came to replacing molars, specialists favored implants as the primary treatment option (*n* = 78, 51.7 percent). GDPs, on the other hand, chose both implants (*n* = 89, 42.6%) and acrylic RPDs (*n* = 87, 41.6%) as their preferred treatment alternatives (*p*=0.004).

#### 3.3.2. Gender

Generally, both male and female practitioners had some background knowledge of the SDA concept. However, female dentists (*n* = 71, 60.7%) seem to replace missing molars more frequently in their practices than male dentists do (*n* = 109, 45.8%) (*p*=0.006). Implants were the first choice for molar replacement for both groups, followed by acrylic RPDs.

#### 3.3.3. UAE Vs Non-UAE Graduates

A significant difference was detected (*p*=0.010) when the application of the SDA concept in practice was compared between UAE and non-UAE graduates. Most UAE graduates did not use the SDA concept in their practice (*n* = 121, 59.9%), while the majority of non-UAE graduates applied it in their practice (*n* = 71, 54.6%).

#### 3.3.4. Years of Experience

A statistically significant association (*p*=0.018) was found when years of clinical experience was compared with awareness of SDA. The awareness of the concept increased with the increase in the years of experience. However, no association was found between the years of experience and applying concept in the clinical practice (*p*=0.118). Most dentists with less than 3 years of experience (*n* = 35, 52.2%) preferred to replace missing molars with acrylic RPD, while implants were the preferred treatment option for dentists with more years of experience, (*p*=0.004).

#### 3.3.5. Type of Dental Practice

A higher percentage of private sector dentists (*n* = 94, 58.8%) chose to replace missing molars, while dentists in the government sector preferred not to replace missing molars (*n* = 108, 56.0%) (*p*=0.004).

### 3.4. Dentists' Opinions Related to Risks and Benefits of the SDA Concept

Great variation was observed in reviewing the dentists' opinions towards the SDA concept regarding appearance, chewing function, speech, and oral comfort. The general opinion among the participating dentists was that there were some risks associated with SDA. Most respondents stated that SDA is associated with teeth migration (*n* = 211, 59.9%), teeth wear (*n* = 196, 55.8%), and/or TMD (*n* = 163, 45.3%). In the evaluation of the advantages associated with SDA, there was a high agreement score for: “simplify oral hygiene,” “allows for simpler treatment planning”, “allows the patient to keep their own natural teeth longer” and “allows better patient economy.” Specialists and GDPs from both genders agreed that SDA contributes to TMDs and teeth migration. Both also disagreed that SDA is associated with any speech problems. Similarly, the comparison between different years of experience with dentists' attitudes towards risks and benefits of SDA showed that as years of experience increases there is a higher agreement that SDA provides better patient economy (*p*=0.04). There was a significant agreement among dentists working in the government and private sectors that SDA provides acceptable chewing function and dental appearance (*p*=0.031, 0.023, respectively).

### 3.5. Dentists' Opinion of Criteria for Proposing the SDA Concept

Most of the responding dentists chose to propose SDA to patients with low economic incomes (*n* = 207, 57.0%), followed by medically compromised and old patients (*n* = 157, 43.3% and *n* = 143, 39.4%).

### 3.6. Dentists' Assessment of Patients' Acceptance of the SDA Concept

A high percentage of dentists reported that they do not propose the SDA concept to their patients (*n* = 165, 46.3%). The patients' response to the suggestion of the SDA as a treatment option were assessed by the respondents as follows: agreed after an explanation was provided (38.5%); agreed immediately (7%); and objections (8.1%). A low percentage of dentists expressed that patients would agree to SDA immediately without an explanation (*n* = 25, 7%).

Years of dental experience showed an association with the reported patient reaction towards SDA (*p*=0.011). Dentists with more than 10 years of clinical experience indicated that their patients agreed to SDA as a treatment option when it was explained to them (*n* = 71, 48.6%), while the majority of dentists with less than 3 years or 3 to 10 years of experience did not propose SDA to their patients, 52.2% (*n* = 35) and 51.1% (*n* = 72), respectively. Although 45.8% (*n* = 160) of the participating dentists, whether working in private or governmental sectors, reported not proposing SDA to their patients, this percentage for private sector dentists (*n* = 85, 53.1%) was significantly higher than government dentists (*n* = 75, 39.7%) (*p* 0.006).

## 4. Discussion

This cross-sectional study surveyed dentists in the UAE with different specialties, backgrounds, and work environments to determine their understanding and application of the SDA concept in their practice.

The 40.3% response rate was in accordance with and, in some cases, higher than the response rates of other similar studies conducted on SDA in the UK (42%) and Australia (40.3%) [14, 20], however, it is considered lower than other studies conducted in KSA, Malaysia, and Jordan, which had response rates of 72.1%, 84%, and 70.7%, respectively [15, 21, 22]. The low response rate could be due to the usage of an online survey distributed via e-mail, as electronic surveys have lower response rates than physical ones [23]. The response rate may also be affected by the nature of the subject matter being investigated, since people who are uninterested in the subject are less likely to respond to the survey.

The majority of dentists in the UAE (65.8%) were aware of the SDA concept, which is comparable to dentists' awareness in a similar study in Australia (61%) [14], but higher than another study in Saudi Arabia (34.4%) [15] and significantly lower than dentists' awareness in Jordan (82.1%) [21]. On the other hand, 34% of respondents were not aware of the SDA concept, which can be considered a high proportion even though the SDA has been described as a viable treatment option in the dental literature for over three decades. Furthermore, among those dentists who were aware of the SDA, the frequency of application was considerably low. The same was found in other studies in various countries [10, 14, 16–19, 24, 25].

Dentists who had graduated from non-UAE countries were more aware of SDA and used it more frequently in their practice than UAE graduates. This can be attributed to the incorporation of the SDA concept in their dental school curriculums. Abu-Awwad et al. [21] reported no link between levels of education and awareness of SDA; in fact, the opposite was shown in this study, with the majority of GDPs learning about SDA only after getting the survey, whilst specialists had prior knowledge. This difference could be due to the fact that dental schools do not include the SDA concept in their undergraduate curricula, and the specialists are first introduced to the concept in their postgraduate studies. Our results also showed that as the number of years of experience increased, so did the level of awareness about SDA. This could be related to the fact that dentists with more years of experience have learned about it through continuing education programs. The finding that dentists with more years of experience were more aware of SDA than dentists with fewer clinical experience contradicts the findings of similar studies in Australia and Jordan, which found that dentists with fewer years of experience were more aware of SDA than dentists with more clinical experience [14, 21]. However, despite the increase in knowledge with advanced years of experience, our study found no link between years of experience and the application of the concept in clinical practice.

GDPs reported replacing missing molars more frequently than specialists, which can be related to the lack of knowledge about SDA among GDPs in the UAE. In the present study, female dentists were more likely to select posterior tooth replacement for SDA patients than male dentists. This result is in agreement with a previous study conducted in Sweden [25]. Fifty percent of the participating dentists voted in favor of replacing missing molars, stating that this will improve the masticatory function and aesthetics of SDA patients. This outcome was in accordance with a previous study conducted in Australia [14], where 77% of participating dentists preferred to replace missing molars.

Implants were the treatment of choice for posterior teeth replacement for most of the participants, followed by acrylic RPDs. In a similar study, dentists in Jordan (84.9%) [21] agreed with UAE dentists on the implant option. Implants have become the trend for the replacement of missing teeth because of their high survival rates and the ability to provide the fixed option that is preferred by most patients [26]. However, dentists in KSA [15] and the UK [27] selected metallic RPD as the preferred treatment modality for SDA cases, while in Tanzania the majority favored using acrylic RPD for SDA cases [17]. Lack of retention and support distally in distal extension RPDs often causes discomfort and dissatisfaction to patients. When compared with RPD, implants provide better occlusal stability, simpler prostheses and more bone preservation [28]. Interestingly, the majority of dentists with less than 3 years of experience (52.2%) preferred to replace missing molars with acrylic RPD, while implants were the selected option for dentists with more years of experience. This could be because the provision of acrylic RPD is a simple, safe nonsurgical option and can be done by dentists with less experience, but with more clinical training and experience, dentists gradually move to implants that require more clinical skills and training.

A high percentage of GDPs preferred to replace missing molars (57.4%), while specialists mostly preferred not to (59.1%). This is in accordance with the fact that specialists showed better awareness of SDA than that of GDPs and, therefore, is more likely to think of SDA as a treatment option. This result is comparable with the results of the Abuzar study in Australia, in which the postgraduate dentists were more aware of the concept than the basic dental degree holders, though the difference was not significant [14].

A higher percentage of private sector dentists (*n* = 94, 58.8%) chose to replace missing molars, while the opposite was found in the government sector (*n* = 108, 56.0%). This can be attributed to the business elements of dental practice interfering with private dentists' decision-making on the replacement of missing molars. Most private dentists are compensated for their work through a commission-based method of payment, where a fee-per-item payment system delivers them a percentage of the fee collected from the patient. Generally, treatment in the government sector is free of charge. This suggests that dentists in the government sector are keen on keeping the patient's own natural teeth for longer periods of time, while the proposal of SDA can have a negative economic impact on dentists working in the private sector.

Generally, the current results showed a positive attitude towards SDA as a treatment option by the participating dentists. Dentists in this study believed that SDA provided acceptable chewing function, dental appearance, oral comfort, and speech. This is comparable with other studies in which dentists agreed that dental arches comprising healthy teeth up to the second premolars can serve satisfactory aesthetics, oral comfort, and function [2, 11, 29]. In the present study, dentists considered SDA a practical treatment option since it can simplify oral hygiene, allow for simpler treatment planning, improved patient economy as well as allowing patients to keep their natural teeth longer. These results are in accordance with other studies that also showed a positive attitude to the SDA concept [11, 14, 18, 27]. Overall, participating dentists considered that there were only a few risks resulting from SDA, such as teeth migration, wear, and TMD. Although the dentists who took part in the study agreed that SDA provides satisfactory chewing function, dental appearance, oral comfort, and speech, they still consider that it is not optimal and that molars should be replaced to improve mastication and aesthetics. This is why half of the participants (*n* = 183, 50.8%) always replace missing molars.

A high percentage of UAE dentists did not propose SDA to their patients (*n* = 165, 46.3%), despite the fact that they were aware of it (*n* = 237, 65.8%). This could indicate that knowing about SDA does not necessarily imply having a thorough understanding of it, which is why most dentists did not consider recommending it to their patients. Clinicians must be able to provide patients with appropriate advice and treatment options, including SDA. Furthermore, it is generally accepted from an ethical standpoint that all treatment options, including no treatment, should be discussed with patients. This highlights the significance of continuing medical education courses and workshops to raise dentists' awareness of this ethical obligation in the UAE. Most dentists with more than 10 years of clinical experience indicated that their patients agreed to SDA as a treatment option when it was explained to them, while most dentists with fewer years of experience did not propose this option. This is likely because dentists with less expertise were less familiar with the concept, or young dentists were more enthusiastic about providing teeth replacement treatment.

Most of the respondents selected to propose SDA to patients with low economic incomes, followed by medically compromised patients. Patients' financial status played a critical role in accepting treatment with SDA as reported by 63% of dentists in a study in Australia and 45.2% of dentists in a study in Malaysia [14, 30]. This seems sensible since providing SDA as an option can reduce the financial burden on the patients as well as reduce the medical risks on medically compromised patients caused by complex restorative treatments, and the subsequent needed maintenance care by both the patient and the clinician. Cost was reported as the main reason to propose SDA to patients in a similar study in Jordan [21]. After receiving sufficient explanation, approximately 38.5 percent of the participating dentists reported that their patients agreed to SDA. This indicates the importance of patients being well-informed and that dentists have effective communication skills.

Some clinical options, such as SDA, are more challenging than others because of controversies, search strategies, and the availability of variable recommendations, as well as the background of clinicians [31]. Generally, SDA provides a less complicated type of treatment that is also less expensive and less time-consuming. Based on the outcomes of this study, it seems that SDA is not being taught in UAE undergraduate dental universities. Its incorporation will provide a more ethical and functional way of treating patients while minimizing the risks on older and medically compromised group of patients, reducing waiting lists for prosthetic rehabilitation cases, and providing an economically positive impact by reducing the chances of overtreatment.

### 4.1. Limitations of the Current Study

Although using an online questionnaire is considered a cost-effective, fast, and simple method that can cover a large group of people, it is associated with certain limitations. These include the inability to explain the questions to the respondents when it is not clear, as well as not being able to control who answers the questions.

In addition, only EMA-registered dentists were invited to participate, which did not necessarily include comparable representatives from various Emirates in the UAE. Therefore, the results cannot be generalized to dentists in the UAE. Lastly, the specialties included in this study include orthodontists and pediatric dentistry specialists, where one can argue that their type of work may not involve rehabilitation of SDA cases. However, in the private sector, many of the specialists are practicing restorative dental treatment along with their own specialty, and this sometimes includes treatment planning of SDA cases.

### 4.2. Future Studies

Further investigations are required to study the effects of different treatment modalities for SDA cases on patient satisfaction and oral health. Surveying patients treated with SDA will provide an adequate level of knowledge on the level of patients' satisfaction and their oral health-related quality of life (OHRQoL).

## 5. Conclusions

Within the limitations of the present study, it can be concluded that UAE dentists are generally aware of the SDA concept and have a positive attitude about it, yet they do not usually apply it in clinical practice. The preferred treatment modality for SDA cases are implants, followed by acrylic RPD.

Dentists in the UAE believe that SDA provides acceptable chewing function, dental appearance, oral comfort, and speech. On the other hand, participating dentists associated SDA with some risks including tooth wear, teeth migration, and TMD.

A good percentage of respondents observed that patients accepted SDA after proper explanation. Furthermore, dentists in this study believe that SDA is a good treatment option for patients with low economic status, followed by medically compromised patients. However, there is a need to increase SDA awareness and acceptance among UAE dentists and patients.

## Figures and Tables

**Table 1:** Characteristics of the sample under study.

Items	No (%)
*Gender*	
Male	239 (66.9)
Female	118 (33.1)

*Year of experience*	
<3 years	67 (18.7)
3–10 years	143 (39.8)
>10 years	149 (41.5)

*Highest qualification*	
GDP	209 (58.1)
Specialist	151 (41.9)

*Specialty*	
Prosthodontics	51 (14.0)
Orthodontics	29 (8.0)
Oral medicine and radiology	2 (0.6)
Pediatric dentistry	26 (7.2)
Endodontics	21 (5.8)
Periodontics	8 (2.2)
Oral surgery	19 (5.2)

*Country of last academic degree*	
UAE	204 (56.2)
Expatriate	131 (36.1)

*Dental practice*	
Government clinic	195 (54.9)
Private clinic	160 (45.1)

**Table 2:** Knowledge about SDA and selected mode of treatment.

Items	No (%)
*Heard about SDA*	
No	123 (34.2)
Yes	237 (65.8)

*Using SDA in practice*	
No	196 (54.7)
Yes, always	14 (3.9)
Yes, rarely	51 (14.20)
Yes, sometimes	97 (27.1)

*Always replacing missing molar*	
No	177 (49.2)
Yes	183 (50.8)

*Treating a patient with missing posterior teeth*	
No treatment	6 (1.7)
Acrylic RPD	129 (35.5)
Cantilever bridge	15 (4.1)
Implant	169 (46.6)
Metallic RPD	44 (12.1)

*Replace missing molar*	
Both (mastication + aesthetics)	159 (44)
To improve aesthetics	2 (0.6)
To improve masticatory ability	160 (44.3)
To satisfy patient's demand	40 (11.1)

## Data Availability

The data used to support the findings of this study are available from the corresponding author upon request.

## Supplementary Materials

Supplementary Materials Appendix A: Questionnaire on SDA awareness, knowledge, and practice of dentists.
