# Supplementary material for: The Shortened Dental Arch Concept: Awareness, Knowledge, and Practice of Dentists in Dubai and the Northern Emirates, United Arab Emirates
Source: Int J Dent. 2022 Nov 2;2022:6018650. doi: 10.1155/2022/6018650 (PMC9646316; doi:10.1155/2022/6018650)
Supplement: Supplementary Materials — Appendix A: Questionnaire on SDA awareness, knowledge, and practice of dentists. [file 6018650.f1.docx]

# Appendix A- Questionnaire on SDA awareness, knowledge, and practice of dentists

1. I agree to participate in the research study. I understand the purpose and nature of this study and I am participating voluntarily. I understand that I can withdraw from the study at any time, without any penalty or consequences.
   - yes
   - No
2. I grant permission for the data generated from this questionnaire to be used in the researcher's publications on this topic.
   - yes
   - No
3. Your gender
   - Male
   - Female
4. Dental Practice
   - Government Clinic
   - Private Clinic
5. Are you a
   - General Dental Practitioner
   - Specialist
6. If you are a specialist, mention your specialty
7. Mention the country of last academic degree taken:
8. Years of experience
   - Less than 3 years
   - 3-10 years
   - More than 10 years
9. Have you heard of the Shortened Dental Arch (SDA)?
   - Yes
   - No
10. Do you use the SDA in your practice?
    - No
    - Yes, rarely
    - Yes, sometimes
    - Yes, always
11. Do you always replace missing molars?
    - Yes
    - No
12. How do you usually treat a patient with missing posterior teeth (Free end)?(you can choose more than one option)
    - Acrylic RPD
    - Metallic RPD
    - Cantilever Bridge
    - Implant(s)
    - No need for treatment
13. Why would you replace missing molars?
    - To improve masticatory ability
    - To improve esthetics
    - Both (mastication + esthetics)
    - To satisfy patient's demand
14. What is your opinion of the SDA regarding the following?

|  | **Satisfactory** | **Acceptable** | **Un-Satisfactory** | **I don’t Know** |
| --- | --- | --- | --- | --- |
| Chewing Function |  |  |  |  |
| Dental Appearance |  |  |  |  |
| Oral Comfort |  |  |  |  |
| Speech |  |  |  |  |

1. SDA contributes to:

|  | **Agree** | **Disagree** | **I don’t Know** |
| --- | --- | --- | --- |
| TMJ Disorders |  |  |  |
| Teeth Wear |  |  |  |
| Teeth Migration |  |  |  |
| Speech Problem(s) |  |  |  |

1. The SDA will:

|  | **Agree** | **Disagree** | **I Don’t Know** |
| --- | --- | --- | --- |
| Simplify oral hygiene for patients |  |  |  |
| Allow better patient economy |  |  |  |
| Allow for simpler treatment planning |  |  |  |
| Allow patient to keep their own teeth longer |  |  |  |
| Reduce the risk of overtreatment |  |  |  |

1. When did you come to know about the SDA?

- More than 10 years back
- 5-10 years
- Less than 5 years back
- Just now

1. What is your patient reaction after you propose the SDA?
   - Objection
   - Agreed immediately
   - Agreed after explanation
   - Didn't propose SDA to my patients
2. In which situations would you propose the SDA? (You can choose more than one option)
   - Caries confined to molars region to
   - Good prognosis of anteriors and premolars
   - Old patient (over 50 years)
   - Limited restorative care
   - Medically compromised patients
   - Financially limited patients
